# Constructing Heterostructured MWCNT-BN Hybrid Fillers in Electrospun TPU Films to Achieve Superior Thermal Conductivity and Electrical Insulation Properties

**DOI:** 10.3390/polym16152139

**Published:** 2024-07-27

**Authors:** Yang Zhang, Shichang Wang, Hong Wu, Shaoyun Guo

**Affiliations:** The State Key Laboratory of Polymer Materials Engineering, Polymer Research Institute of Sichuan University, Chengdu 610065, China; 17844626708@163.com (Y.Z.); 15166793239@163.com (S.W.); nic7702@scu.edu.cn (S.G.)

**Keywords:** electrostatic self-assembly, MWCNT-BN hybrids, thermal conductivity, thermal resistances, electrical insulation

## Abstract

The development of thermally conductive polymer/boron nitride (BN) composites with excellent electrically insulating properties is urgently demanded for electronic devices. However, the method of constructing an efficient thermally conductive network is still challenging. In the present work, heterostructured multi-walled carbon nanotube-boron nitride (MWCNT-BN) hybrids were easily prepared using an electrostatic self-assembly method. The thermally conductive network of the MWCNT-BN in the thermoplastic polyurethane (TPU) matrix was achieved by the electrospinning and stack-molding process. As a result, the in-plane thermal conductivity of TPU composite films reached 7.28 W m^−1^ K^−1^, an increase of 959.4% compared to pure TPU films. In addition, the Foygel model showed that the MWCNT-BN hybrid filler could largely decrease thermal resistance compared to that of BN filler and further reduce phonon scattering. Finally, the excellent electrically insulating properties (about 10^12^ Ω·cm) and superior flexibility of composite film make it a promising material in electronic equipment. This work offers a new idea for designing BN-based hybrids, which have broad prospects in preparing thermally conductive composites for further practical thermal management fields.

## 1. Introduction

With the increased integration and high power density of electronic devices, military equipment, and communication instruments, heat accumulation continues to be a critical limiting factor in the narrow space [1,2,3]. Highly efficient heat dissipation is essential to ensure the service life and stability of the electronic components. Polymer-based thermally conductive materials have been extensively investigated due to their cost-effectiveness, lightweight, and ease of processing [4,5,6]. Nevertheless, the polymer materials are limited in practical applications because they possess a low intrinsic thermal conductivity (TC, <0.50 W m^−1^ K^−1^) [7,8,9]. In the past decades, aluminum nitride [10], alumina [11], boron nitride [12,13,14,15], silicon carbide [16], metal [17], carbon nanotube [18,19], and graphene [20,21] have served as thermally conductive fillers to enhance the TC of polymer materials. Particularly, BN has attracted tremendous attention in the development of thermally conductive yet electrically insulating composites because of its extremely high thermal conductivity (400 W m^−1^ K^−1^) and relatively wide band gap (5.5–6.4 eV) [22,23,24]. However, due to its chemical inertness, the improvement of the obtained TC is usually limited due to the undesirable dispersion of BN in polymer composites [25].

Interfacial optimization has become a trend through the surface treatment of BN to improve the TC of polymer-based materials. The common way is to modify the BN surface through covalent bonding and noncovalent interactions, which could promote the BN dispersion and the phonon transfer from the BN to the polymer matrix, and thus enhance the TC of polymer composites [26,27,28,29]. For example, Liu et al. prepared (3-aminopropyl) triethoxysilane (APTES) functionalized BN, and the epoxy/BN composites reached 5.86 W m^−1^ K^−1^ at a filler content of 40 wt% [30]. Additionally, Liu et al. functionalized a noncovalent BN with polydopamine, which could improve interfacial compatibility and uniform dispersion of BN in the polymer matrix [31]. Although the thermal conductivity can be effectively enhanced by optimizing the dispersion of BN, it is still a challenge to further increase thermal conductivity by constructing an efficient thermally conductive network of polymer composites.

Previous studies have demonstrated that hybrid thermally conductive fillers prepared with different dimensions and morphologies have been widely used to fabricate multifunctional polymer composites, which also provide a practical method for constructing efficient thermally conductive networks of polymer composites [32,33,34,35,36,37]. Silver nanoparticles (AgNPs) are introduced onto the surface of BNNS to construct efficient thermally conductive pathways, resulting in a higher TC of the composite [38]. Similarly, Qian et al. developed a novel hetero-structured BN/ZC hybrid filler through in-situ growth of CNTs onto the surface of BNNSs, and thus the TC of epoxy composites reached 3.21 W m^−1^ K^−1^ at a filler content of 9.86 vol%, which is increased by about 13.4 times compared to that of pure epoxy [39]. As a result, it is found that the hybrid thermally conductive fillers can construct more thermal conduction pathways, ultimately improving the TC of thermally conductive composites.

Furthermore, it is known to all that the TC of the composite can be further enhanced by the orientation of single or multi-hybrid fillers with anisotropic properties among the polymer matrix [40,41,42,43]. The commonly used strategies are spin-coating [30], solvent-casting [44,45], hot-pressing [46,47], vacuum filtration [48,49], electrospinning [50,51], etc. Recently, advanced functional composites based on electrospinning have attracted much attention. It has also been found that the anisotropic thermally conductive fillers tend to be oriented along the nanofiber, which can help to achieve higher TC. Shen et al. also aligned BNNS in a horizontal direction by electrospinning technology to build efficient thermal conduction pathways, and the obtained composite possessed a high in-plane TC of 10.9 W m^−1^ K^−1^ [52]. Accordingly, it can be concluded that combining the orientation and the hybrid thermally conductive fillers can enhance the TC of the composites effectively.

In this study, one-dimensional polyethyleneimine (PEI) modified multi-walled carbon nanotubes (MWCNTs) were successfully introduced onto the polydopamine (PDA) functionalized BN via the electrostatic self-assembly method. Then, the obtained MWCNT-BN hybrid fillers were oriented along the fiber during the electrospinning and hot-pressing processes. The MWCNT-BN hybrid fillers significantly construct efficient thermal conduction pathways compared to single BN fillers, displaying superior TC enhancement in TPU composite films. Furthermore, it is worth noting that the addition of a low concentration of MWCNTs can not only greatly enhance the TC of the composite but also maintain electrical insulation properties. The excellent TC, electrical insulation, and mechanical properties of composite ensure its great potential applications in electronic devices, such as computers and drones.

## 2. Materials and Methods

### 2.1. Materials

TPU (Elastogran S85A) was bought from BASF Co., Ltd. (Shanghai, China). MWCNTs-COOH were obtained from Chengdu Organic Chemicals Co., Ltd. (Chengdu, China). The MWCNTs-COOH (MWCNT) had the following features: a length of 10−30 μm and a diameter of 5–15 nm. Hexagonal BN (average particle size of 1 µm) was obtained from Yingkou Tianyuan Chemical Research Institute Co., Ltd. (Hubei, China). Tris (hydroxymethyl) aminomethane (Tris), dopamine hydrochloride, and PEI (Mw = 1800) were purchased from Adamas Reagent Co., Ltd. (Shanghai, China). Ethanol, N,N-dimethylformamide (DMF), and tetrahydrofuran (THF) were purchased from Chengdu Kelong Chemicals Co. Ltd. (Chengdu, China).

### 2.2. Preparation of MWCNT-BN Hybrids

#### 2.2.1. Preparation of PDA Modified BN (PDA-BN)

Typically, 2.0 g of BN powder was dispersed in 400 mL of tris-buffer solution (10 mM, pH 8.5) by ultrasonic for 1 h, then 0.8 g of dopamine hydrochloride was added and stirred for 12 h at ambient temperature, followed by vacuum-assisted filtration, washing, and drying to obtain BN-PDA. 

#### 2.2.2. Preparation of PEI Functionalized MWCNT (PEI-MWCNT)

Firstly, 5 g of PEI was added to deionized water (10 mg/mL) and this was stirred at room temperature until the PEI was completely dissolved. Subsequently, 0.5 g of MWCNTs were added into the PEI solution and it was ultrasonically processed for 1 h and stirred at 80 °C for another 12 h. Finally, the PEI-MWCNT was filtered, washed five times, and dried at 80 °C overnight.

#### 2.2.3. Preparation of MWCNT-BN Hybrids

**Figure 1:**
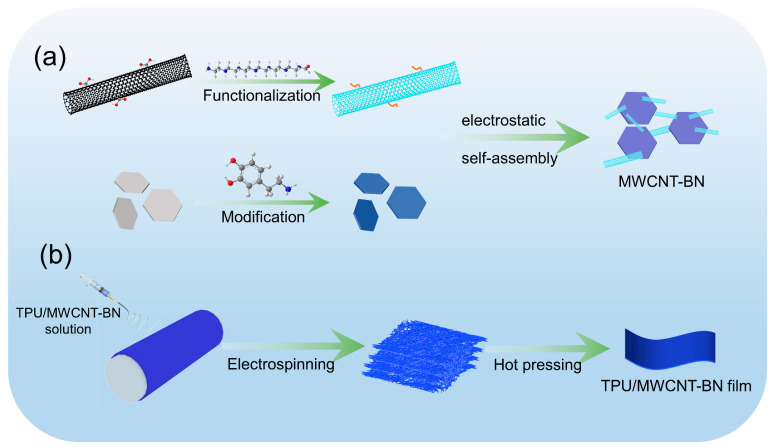
(**a**) Schematic illustration of fabrication for (**a**) hetero-structured MWCNT−BN fillers and (**b**) thermally conductive TPU/MWCNT−BN composite films.

According to Figure 1a, 1.2 g of BN-PDA was dispersed in 100 mL of deionized water for 0.5 h by ultrasound, and, at the same time, a certain mass of PEI-MWCNT was dispersed in 50 mL of deionized water. Then, the PEI-MWCNT dispersion was gradually added to the BN-PDA dispersion and it was stirred at room temperature for 12 h. Finally, the product was centrifuged, washed, and dried to obtain MWCNT-BN hybrids.

### 2.3. Fabrication of the TPU/MWCNT-BN Composite Films

According to Figure 1b, a certain mass of MWCNT-BN hybrids was dispersed in a mixed solvent of DMF and THF (1:1 by weight) under an ultrasonic process for 0.5 h. TPU was added to the above solution until it was completely dissolved. Then, the electrospinning process was carried out according to our previous work. Finally, the TPU/MWCNT-BN electrospun fibers were hot pressed at 170 °C and 20 MPa for 10 min to obtain TPU/MWCNT-BN composite films. For comparison, the pure TPU film and TPU/BN composite films were also fabricated through the same procedures.

### 2.4. Characterization

The surface morphology of BN, PDA-BN, MWCNT-BN, and the electrospun fibers and the cross-section of the composite films were observed by scanning electron microscopy (SEM, FEI Quanta 250, Hillsboro USA). The morphologies of the TPU fibers, TPU/BN fibers, and TPU/MWCNT-BN fibers were observed by transmission electron microscopy (TEM, FEI Tecnai G2 F20, Hillsboro USA) at 200 kV. Atomic force microscopy (AFM, AFM+, Anasys Instruments, Hillsboro USA) images were recorded to measure the thickness of BN. Fourier transform-infrared (FT-IR) spectroscopies of MWCNT and PEI-MWCNT were obtained using the FT-IS10 spectrometer (FT-IR, Thermo Fisher Scientific, Massachusetts USA) over a frequency range of 400–4000 cm^−1^. Raman spectroscopies of BN, PDA-BN, MWCNT, PEI-MWCNT, and MWCNT-BN were obtained using a micro-Raman spectrometer (InVia Reflex, London UK) with laser light focusing at a wavelength of 532 nm. The crystal structures of composite films were characterized by X-ray diffraction (XRD, Ultima IV, Rigaku, Tokyo Japan) in the 2θ range of 2–90°. The chemical compositions of BN, PDA-BN, MWCNT, PEI-MWCNT, and MWCNT-BN were determined by X-ray photoelectron spectroscopy (XPS, AXIS Ultra DLD (Kratos) Kyoto Japan). Thermal gravimetric (TG) analyses were performed using TG209F1 equipment (Selb Germany) under a nitrogen atmosphere at a heating rate of 10 °C/min. A Zetasizer Nano ZS analyzer (Malvern Instruments Ltd., Malvern, UK) was used to characterize the zeta potentials of BN, PDA-BN, MWCNT, PEI-MWCNT, and MWCNT-BN at pH = 7.0. The in-plane or through-plane TC of composite films was calculated by multiplication of thermal diffusivity (α, mm^2^/s), density (ρ, g/cm^3^), and specific heat capacity (Cp, J/(g·K)), i.e., TC = α × Cp × ρ. A laser flash apparatus (LFA, NETZSCH LFA 467, Selb Germany) was used to measure the α of composite films at 25 °C. The water displacement method was performed to determine the ρ of composite films. Differential scanning calorimetry (DSC, TA Instruments, New Castle, DE, USA) was used to measure the Cp of composite films at 25 °C. An infrared camera (T620, FLIR Systems Inc., Boston, MA, USA) recorded the variations in the surface temperature of the LED device. A high resistance meter (KEITHLEY, 6487 Oregon, OR, USA) was carried out to measure the volume electrical resistivity of the composite films. The tensile strength at the breaking points of the composite samples was determined using an INSTRON 5966 (Shanghai China) electronic tensile strength meter with a tensile rate of 200 mm/min.

## 3. Results and Discussion

### 3.1. Characterization of MWCNT-BN Hybrids

**Figure 2:**
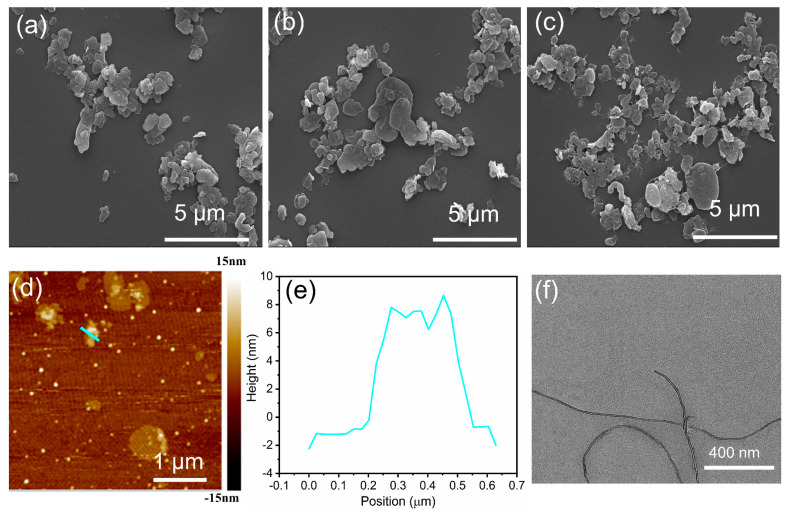
SEM images of (**a**) BN, (**b**) PDABN, and (**c**) MWCNTBN. (**d**) AFM image and (**e**) height profile of the BN. (**f**) TEM image of MWCNT.

Bulk BN displays a similar multi-layered structure to graphite and is also a promising thermally conductive filler [50], as shown in Figure 2a. First, BN is easily and successfully modified through dopamine chemistry, which effectively improves the chemical activity of inert BN. As seen in Figure S1, the color of the BN aqueous solution changes from white to gray after modification because of the formation of a PDA layer on the BN surface. At the same time, the dispersibility of PDA-BN is also significantly improved compared to BN after modification. Figure 2b shows the SEM image of PDA-BN with an average size of 0.47 μm (Figure S2), and the dimension has not changed compared to the pure BN. The MWCNT-BN hybrid is obtained by the electrostatic self-assembly method. The MWCNT shows the uniform size and high aspect ratio in Figure 2f. The AFM image and the corresponding thickness curve of BN are shown in Figure 2d,e, and the thickness is about 10 nm. Thermally conductive pathways can be easily constructed using the electrostatic self-assembly method due to the different dimensions and morphologies of BN and MWCNT. As can be seen from Figure 2c, the MWCNT with a high aspect ratio is anchored on the surface of BN without agglomerating and can act as an effective “bridge” to significantly reinforce the thermal conduction pathways (Figure S3).

**Figure 3:**
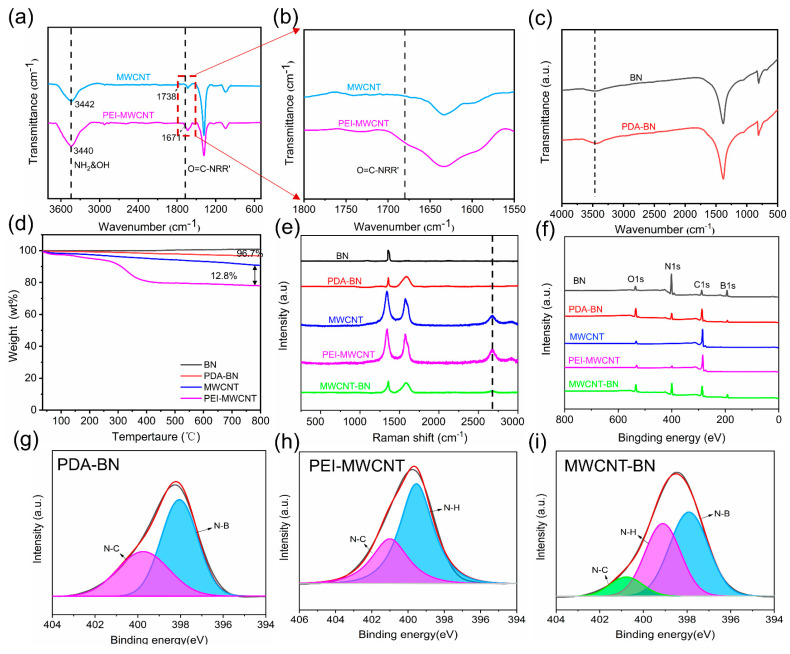
(**a**,**b**) FT-IR spectra of MWCNT and PEI−MWCNT. (**c**) FT-IR spectra of BN and PDA−BN. (**d**) TGA spectra of BN, PDA−BN, MWCNT, and PEI−MWCNT. (**e**) Raman results of BN, PDA−BN, MWCNT, PEI−MWCNT, and MWCNT-BN. (**f**) XPS patterns of BN, PDA-BN, MWCNT, PEI−MWCNT, and MWCNT−BN. (**g**–**i**) N1s XPS core level of PDA−BN, PEI−MWCNT, and MWCNT−BN.

FTIR measurement is carried out to prove effective modification of BN and MWCNT. Compared to MWCNT, a small new peak at 1671 cm^−1^ is assigned to O=C-NRR′C=O stretching vibration in the amide I band or C=C skeletal vibration. The absorbance peaks at 3440 cm^−1^ are attributed to the -NH and -OH groups, which are much stronger than MWCNT, indicating that PEI is successfully grafted onto the surface of MWCNT (Figure 3a,b). Obviously, the dispersibility of MWCNT and PEI-MWCNT is significantly improved compared with CNT after modification (Figure S4). As shown in Figure 3c, the absorption peaks at 811 and 1376 cm^−1^ are attributed to the in-plane stretching vibration and out-of-plane bending vibration of the B−N bond, respectively [51]. After PDA modification, the peak corresponding to oxygen-containing groups becomes stronger, suggesting the successful preparation of PDA-BN. The successfully modified BN and MWCNT are further validated using XPS analysis (Figure 3f–i). From Figure 3f, the peak intensities of C 1s and O 1s for PDA-BN are remarkably increased compared with pristine BN, demonstrating that the PDAs are successfully coated on the surface of BN (Table S1). Furthermore, the N 1s spectrum of BN is deconvoluted into 398.25 eV, corresponding to the N-B bond (Figure S5). In comparison, a new signal of 399.8 eV occurs in the N 1s spectrum of PDA-BN, which is assigned to the existence of N-C, further indicating that DA is successfully polymerized on the surface of BN (Figure 3g). The two characteristic peaks at 532.2 eV (O 1s) and 284.9 eV (C 1s) are detected for MWCNT, while a new peak of N 1s for PEI-MWCNT exists at 399.3 eV, which is fitted to 399.5 eV (N-H) and 401.0 eV (N-C) due to the PEI being grafted onto the surface of MWCNT (Figure 3h). As presented in Figure 3i, it is also observed that the N 1s peak of MWCNT-BN is deconvoluted into three peaks, N-C (400.8 eV), N-H (399.1 eV), and N-B (397.9 eV), demonstrating the successful preparation of MWCNT-BN. 

Furthermore, the TGA curves in Figure 3d show that the content of the PDA layer is calculated at 3.3%. Additionally, the PEI grafted on the surface of MWCNTs is about 12.8%. As shown in Figure S6, the zeta potential of BN is estimated to be −32.8 mV due to the hydroxyl groups attached to the surface of BN, resulting in a negatively charged state. Meanwhile, the zeta potential of PDA-BN is about −37.2 mV, mainly because more hydroxyl groups are generated on the surface of BN, which is consistent with the results of FTIR measurement. In addition, the zeta potential of MWCNT in deionized water is −41.0 mV, indicating the impossible self-assembly with the PDA-BN that has a negative charge. Thus, the PEI is successfully grafted on the surface of MWCNT and the resultant PEI-MWCNT displays a zeta potential value of 33.3 mV. The photographs of PDA-BN before and after mixing with PEI-MWCNT are shown in Figure S7. The PDA-BN exhibits a homogeneous dispersion in deionized water, while the PDA-BN fillers quickly precipitate to the bottom due to the strong electrostatic adsorption between them. Raman spectra analyses are performed to further confirm the surface chemical compositions of fillers (Figure 3e). The characteristic peak at 1358 cm^−1^ is ascribed to high-frequency intralayer E2g tangential mode for BN. A new signal at 1587 cm^−1^ occurs after functionalization, which is attributed to the deformation of catechol from PDA. Furthermore, due to the PDA layer attached to the BN surface, the intensity of the BN peak at 1358 cm^−1^ is merely weakened. The MWCNT has three characteristic peaks, i.e., D peak (1343 cm^−1^), G peak (1575 cm^−1^), and G’ peak (2678 cm^−1^). The G peak is caused by the vibration of the sp_2_ carbon atom in the carbon nanotubes, and its location and intensity are closely related to the structure and chirality of the carbon nanotubes. The intensity of the G peak is high, generally occurring at about 1500 cm^−1^. The D peak is due to disordered vibrations in carbon nanotubes and usually occurs at about 1300 cm^−1^. The intensity of the D peak is low, but it is related to the diameter, length, and chirality of the carbon nanotubes. The G’ is caused by the Van der Waals force between the layers of carbon nanotubes, which occurs at about 2000 cm^−1^. The G’ peak intensity is low and sensitive to the chirality, diameter, and number of layers of carbon nanotubes. Meanwhile, the PEI-MWCNT has the same peak and intensity as MWCNT, indicating that the PEI has little impact on the crystal structure of MWCNT. Due to the small amount of PEI-MWCNT in the MWCNT-BN hybrid filler, the D and G peaks of PEI-MWCNT are not easily observed, while a weak G’ peak can be observed in the Raman spectra of MWCNT-BN, further proving the successful preparation of MWCNT-BN.

### 3.2. Preparation and Morphology of TPU/MWCNT-BN Composite Films

**Figure 4:**
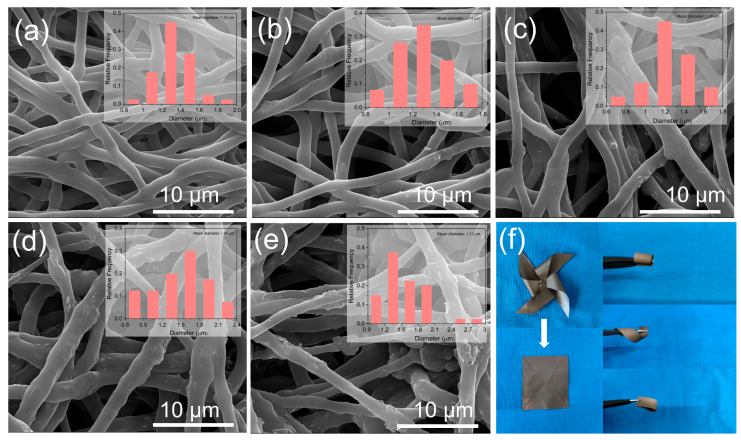
(**a**–**e**) SEM images of TPU/MWCNT-BN fibers with different filler content of 0 wt%,10 wt%, 20 wt%, 30 wt%, and 40 wt%. The insets in (**a**–**e**) represent the diameter distribution of fibers. (**f**) Photographs of the TPU/MWCNT-BN composite films.

The SEM images of pure TPU and TPU/MWCNT-BN electrospun fibers are shown in Figure 4. The pure TPU fibers exhibit a smooth and uniform surface, illustrating that TPU can be completely dissolved in the DMF/THF solution (Figure 4a). The average diameter of TPU fibers is about 1.29 μm. With the MWCNT-BN concentrations rising, the average diameter of the electrospun fibers increases gradually. The primary reason is that the introduction of MWCNT-BN fillers increases the viscosity of the electrospinning solution. Meanwhile, due to the instability of the jet, the uniformity of the fiber diameter decreases and the MWCNT-BN also occurs on the surface of electrospun TPU/MWCNT-BN fibers. Figure S8 shows the TEM images of TPU, TPU/40 BN, and TPU/40 MWCNT-BN electrospun fibers, respectively. There are no fillers observed in pure TPU fiber, while the BN is better dispersed in the TPU fibers. Furthermore, the heterostructured MWCNT-BN is also oriented in the TPU fibers, which is better for forming thermal conduction pathways than BN. The cross-sectional morphologies of the TPU/MWCNT-BN composite film after hot-pressing are shown in Figure S9. It can be clearly observed that the MWCNT-BN exhibits obvious orientation along the in-plane direction. To visually demonstrate the flexibility of TPU/40MWCNT-BN composite films, Figure 4f shows the optical photographs of TPU/MWCNT-BN composite films, which could be bent, folded, curled, and fixed into the shape of a small windmill without obvious cracks. Meanwhile, the composite film could withstand the pulling of 500 g without any breakages, demonstrating its application prospects in electronic equipment. 

### 3.3. Properties of the TPU/MWCNT-BN Composite Films

**Figure 5:**
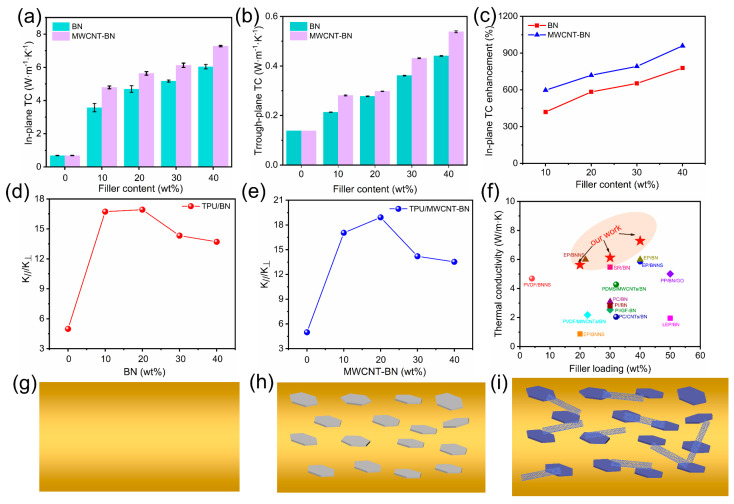
(**a**) In-plane TC and (**b**) through-plane TC of TPU/BN and TPU/MWCNT−BN composite films with different filler content. (**c**) The in-plane TC enhancement of TPU/BN and TPU/MWCNT−BN composite films with different filler content. (**d**,**e**) Thermal conductivity anisotropy of TPU/BN and TPU/MWCNT−BN composite films, respectively. (**f**) Comparison of in-plane TC of TPU/MWCNT−BN composite films and other thermally conductive composite reported previously. (**g**–**i**) Schematic diagram of thermally conductive pathways of TPU film, TPU/BN composite film and TPU/MWCNT−BN composite film.

Figure 5 shows the in-plane TC and out-of-plane TC of TPU/BN and TPU/MWCNT-BN composite films with different content, respectively. From Figure 5a, pure TPU film shows a low in-plane TC of 0.68 W m^−1^ K^−1^ due to phonon scattering between the interfaces, defects, and impurities [52]. It is evident that the introduction of BN can sharply enhance the in-plane TC of TPU/BN composite film. Furthermore, as the BN content increases, the in-plane TC of TPU/BN composite films shows a notably increasing tendency. The TPU/40 BN composite film has an excellent in-plane TC of 6.04 W m^−1^ K^−1^, which is higher than that of pure TPU. This is mainly because the BNs are mostly arranged along the horizontal direction of the composite. In addition, it is worth noting that the TPU/MWCNT-BN composite films exhibit higher thermal conductivity compared with TPU/BN composite films at the same filler content, which is primarily due to the fact that the heterogeneous MWCNT-BN is more prone to constructing continuous thermally conductive pathways than BN. One can see that the TPU/40 MWCNT-BN composite film shows ultrahigh in-plane TC of 7.28 W m^−1^ K^−1^, which is 20.5% higher than the TPU/40 BN composite film. Each composite film displays a much lower out-of-plane TC, as shown in Figure 5b. The through-plane TC of TPU/40 MWCNT-BN composite film increases to 0.54 W m^−1^ K^−1^ when the mass fraction of MWCNT-BN is 40 wt%, which is 3.86 times larger than that of pure TPU film (0.14 W m^−1^ K^−1^). Similarly, the through-plane TC of TPU/MWCNT-BN composite films is also higher than that of TPU/BN composite films, the through-plane TC value of TPU/40 BN composite film is as high as 0.44 W m^−1^ K^−1^.

Due to the typical anisotropic feature of h-BN, the orientation of BN in the TPU matrix can be studied by the XRD investigation, which is a vital factor in improving the TC of the composite. The (002) plane and (100) plane of BN are assigned to the BN oriented in in-plane and through-plane directions, respectively. Therefore, the in-plane orientation degree of BN in the TPU matrix can be expressed by the intensity ratio I (002)/I (100). As presented in Figure S10, the peak intensities of (002), (100), and (004) increase as the MWCNT-BN content increases. The high I (002)/I (100) peak ratio was obtained in the TPU/MWCNT-BN composite films, indicating that the MWCNT-BN displays a well-oriented structure in the TPU matrix because of the electrospinning and hot-pressing methods, which could greatly enhance the in-plane TC of TPU composite films. 

In addition, the TC enhancement (TCE) is calculated to compare the in-plane TC of TPU composite films with that of pure TPU films, which is defined as follows:

(1) $$TCE=\frac{TC-{TC}_{0}}{{TC}_{0}}$$

where TC and TC_0_ are the in-plane TC of TPU composite films with different filler content and pure TPU film, respectively. As shown in Figure 5c, the TCE value of TPU/40BN composite film is as high as 778.6% when the BN content reaches 40%. In contrast, at the same filler content, the TCE value is greatly enhanced to 959.4% for TPU/40MWCNT-BN, indicating that the MWCNT-BN shows better TC enhancement than BN. Furthermore, the anisotropic TC of the composite films is also studied. It is found that the in-plane TC of TPU/BN and TPU/40MWCNT-BN is higher than that of the through-plane TC, displaying a high anisotropic index (AI, the ratio values of K∥ and K⊥), as shown in Figure 5d,e. This is due to the well-dispersed BN and MWCNT-BN which are highly oriented along the in-plane direction because of the synergy effect of electrospinning and hot pressing method, reducing the phonon scattering in this direction. Figure 5f and Table S2 summarize the previously reported in-plane TC of BN-based thermally conductive materials. Compared with other polymer materials, TPU/MWCNT-BN composite films exhibit considerable competitiveness. The in-plane TC of TPU/40MWCNT-BN composite film is up to 7.28 W m^−1^ K^−1^, which is greater than most thermally conductive materials previously reported. The superior in-plane TC is due to the formation of heterostructured MWCNT-BN hybrid fillers as well as the oriented microstructure of the composite films. To further make sense of the possible mechanism of enhanced thermal conductivity, the thermal conduction pathways in the fibers are graphically displayed in Figure 5g–i.

Due to the severe phonon scattering between the interfaces, defects, and impurities, thermal conduction pathways are difficult to construct in pure TPU, which exhibits a low value of TC. When BN is dispersed into the TPU matrix, the stacked BN in the TPU fibers is conducive to phonon transfer, however, it is not enough to construct continuous thermal conduction pathways. Nevertheless, heterostructured MWCNT-BN hybrid fillers more easily form highly efficient phonon transmission pathways. Therefore, phonons can be efficiently transported in the TPU fibers, resulting in a significantly improved TC.

**Figure 6:**
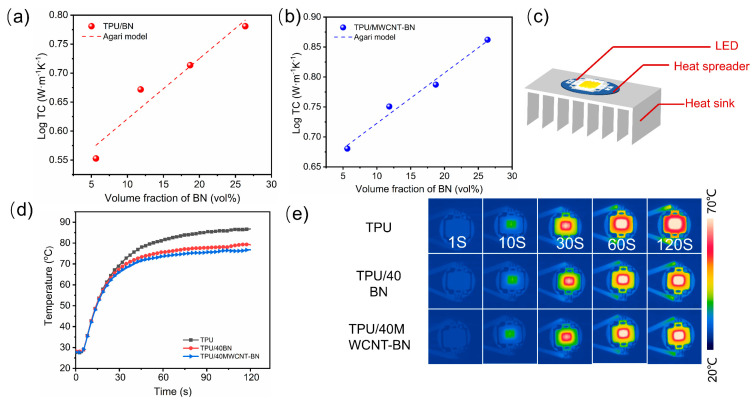
Agari model fitting curve of composite films: (**a**) TPU/BN and (**b**) TPU/MWCNT−BN. (**c**) Schematic illustration of the thermal conduction in an LED bulb. (**d**) Surface temperature versus time of the LED chip when TPU, TPU/40BN, and TPU/40MWCNT−BN as a heat spreader between LED chips and the heat sink and (**e**) corresponding IR images.

The Agari model is employed to elucidate the effects of MWCNT on constructing thermal conduction pathways in TPU composite films. Generally, the in-plane TC of composite films can be effectively predicted by the following equation:

(2) $$\log K_{C}=V_{f}C_{2}logK_{f}+\left(1-V_{f}\right)log(C_{1}K_{m})$$

where K_c_, K_m_, and K_f_ are the TCs of the composite films, matrix, and fillers, respectively; K_f_ is the volume fraction of fillers, C_1_ represents that the fillers influence the polymer crystallinity; C_2_ suggests that the ability of the fillers to form thermal conduction pathways, 0 ≤ C_2_ ≤ 1. Figure 6a,b show the fitted curves of TPU/BN and TPU/MWCNT-BN composite films, respectively. It is worth noting that the obtained C_2_ value of TPU/BN composite films is 0.248, while a higher C_2_ value is obtained (0.287) for TPU/MWCNT-BN composite films, indicating that the MWCNT more easily forms efficient heat conduction pathways (Table S3). In addition, the Foygel model is further fitted to calculate the interfacial thermal resistances (R) of TPU composite films. The fitting curves and calculation results of the TPU composite films are shown in Figure S11 and Table S4, and the calculation process is exhibited in detail in the supporting information. The interfacial thermal resistances of TPU/BN and TPU/MWCNT-BN are 1.17 × 10^6^ K/W and 7.43 × 10^6^ K/W, showing a decrease of 36.5% due to the addition of MWCNT. Therefore, the fitting results of the Foygel model illustrate that the preparation of MWCNT-BN hybrids is conducive to reducing the R-value of TPU composite films.

To further demonstrate the potential application of composite films as thermal management materials in electronic devices, pure TPU film, TPU/40 BN composite film, and TPU/40 MWCNT-BN composite film with the same size are sandwiched between a light-emitting diode (LED) chip and a heat sink, respectively (Figure 6c). Surface temperature variations of the LED chip with a working time of 120 s are recorded by the infrared thermal imager. As shown in Figure 6d, the surface temperature of the LED chip on TPU/40 BN and TPU/40 MWCNT-BN composite films increased to 79.2 °C and 76.8 °C, while that of pure TPU film reached as high as 86.7 °C. It is worth noting that the LED chip shows the slowest heating rate when the TPU/40 MWCNT-BN composite film is sandwiched between an LED chip and a heat sink (Figure 6e). The infrared thermal image results also verify a better thermal conduction ability of TPU/40 MWCNT-BN composite film than TPU film and TPU/40 BN composite film. Therefore, the flexible TPU/40 MWCNT-BN composite films have great potential in the field of electronic components to ensure service life and stability.

**Figure 7:**
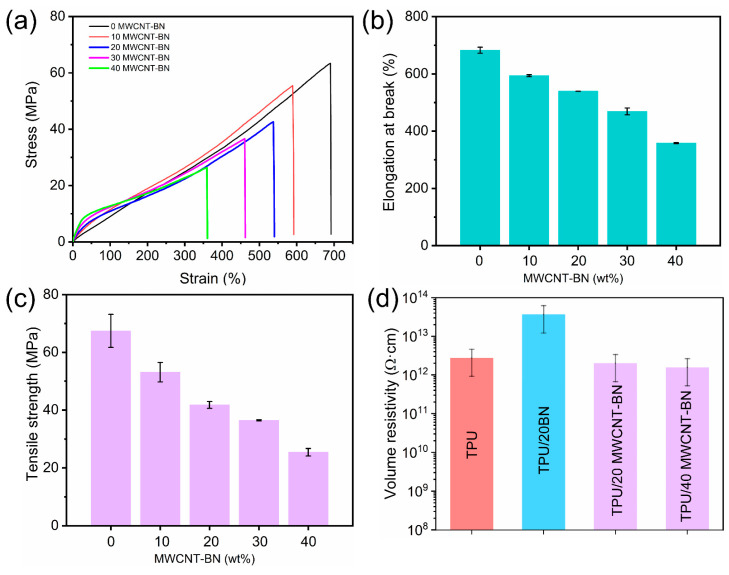
(**a**) Tensile stress–strain curves of the TPU film and TPU/MWCNT-BN composite films. (**b**) Elongation at break and (**c**) tensile strength of TPU/MWCNT-BN composite films. (**d**) Volume resistivity of TPU, TPU/BN, and TPU/MWCNT-BN composite films.

In addition to high TC, the excellent electrical insulation property is also important for thermal management applications to ensure the efficient operation and safety of electronic equipment. Figure 7d shows the volume resistivity results of pure TPU, TPU/BN, and TPU/MWCNT-BN composite films. The volume resistivity of TPU/20 BN composite film is higher than pure TPU film, which is mainly due to the introduction of BN with ultra-high resistivity. Nevertheless, the TPU/MWCNT-BN composite films exhibit lower volume electrical resistivities because of the addition of MWCNT. It is found that the volume resistivity of TPU/40 MWCNT-BN composite film is as high as 2 × 10^12^ Ω·cm, which is far beyond the standard of insulating materials (10^9^ Ω·cm). The TPU/40 MWCNT-BN composite film is connected to an LED in series under a 3 V external voltage (Figure S12). It can be seen that the LED lamps failed to light up when integrated with the TPU/40 MWCNT-BN composite film, further visually proving the insulating property of the composite films. Therefore, it is believed that the composite films can be satisfied with the fields of high electrical insulation requirements. The TGA curves of TPU film and TPU/MWCNT composite films are shown in Figure S13. It is found that both the TPU film and TPU/MWCNT-BN composite films show excellent thermal stability. Moreover, with an increasing MWCNT-BN content, the decomposition temperatures of 10 wt% (T_10%_) and 5 wt% (T_5%_) are enhanced, suggesting that the thermal stability of the composite film is increased. The mechanical properties of TPU/MWCNT-BN composite films are characterized, and the results are shown in Figure 7a. The tensile strength and elongation at the break of pure TPU film are 67.4 MPa and 682.9%, respectively. It can be clearly observed that the tensile strength and elongation at the break of composite films decrease gradually with increasing MWCNT-BN loadings (Figure 7b,c). In general, more interfaces between the thermally conductive filler and the polymer matrix are formed, which act as stress concentration points, leading to the decline in the mechanical properties of composite films. A tensile strength of 25.44 MPa is obtained when the MWCNT-BN loading is up to 40 wt%. At the same time, the composite films maintain superior flexibility, which is also vital in the field of electric equipment and electronic devices.

## 4. Conclusions

In conclusion, heterostructured MWCNT-BN hybrid fillers were prepared via a facile electrostatic self-assembly method, and highly thermally conductive and electrically insulating TPU/MWCNT-BN composite films were successfully fabricated by electrospinning and hot-pressing. The positively charged MWCNT was anchored on the surface of BN and served as a bridge to connect the uncontacted BN, endowing the TPU/40 MWCNT-BN composite film with a high in-plane TC of 7.28 W m^−1^ K^−1^, which is an increase of 959.4% compared to pure TPU films. Moreover, infrared thermal images demonstrated that the TPU/40 MWCNT-BN composite film exhibited strong heat dissipation capability for LED lamps. Furthermore, the composite film possessed superior heat resistance, outstanding electrical insulation properties (about 10^12^ Ω·cm), and perfect flexibility, indicating that it can be widely used as a thermal interface material in high power density electrical equipment and electronic devices.

## Data Availability

Data from this study are available upon request from the corresponding authors.

## Supplementary Materials

The following supporting information can be downloaded at: https://www.mdpi.com/article/10.3390/polym16152139/s1, Figure S1: Optical images of BN and PDA-BN.; Figure S2: Lateral size statistics of BN, PDA-BN and MWCNT-BN. Figure S3: SEM image of MWCNT-BN. Figure S4: Optical images of CNT, MWCNT and PEI-MWCNT. Figure S5: N1s XPS core level of BN and MWCNT. Figure S6: Zeta potential of BN, PDA-BN, MWCNT, PEI-MWCNT and MWCNT-BN. Figure S7: Optical images the fabrication process of MWCNT-BN. Figure S8: TEM images of (a) TPU, (b) TPU/40BN and (c) TPU/40MWCNT-BN electrospun fibers, respectively. Figure S9: Cross-sectional morphologies of the TPU/40MWCNT-BN composite film. Figure S10: XRD patterns of TPU/MWCNT-BN composite film with different filler content. Figure S11: Foygel model fitting curves of composite fims: (a) TPU/BN; (b) TPU/MWCNT-BN. Figure S12: Insulation exhibition of TPU/40MWCNT-BN compsoite films. Figure S13: TGA curves of TPU/MWCNT-BN composite film with different filler content. Table S1: Atomic weight ratio of BN, PDA-BN, MWCNT, PEI-MWCNT and MWCNT-BN; Table S2: Comparison of in-plane TC of TPU/MWCNT-BN composite films and other thermally conductive composite reported previously. Table S3: Agari model fitting parameters of composite films. Table S4: Foygel model fitting parameters of composite films.
