# Supplementary material for: Constructing Heterostructured MWCNT-BN Hybrid Fillers in Electrospun TPU Films to Achieve Superior Thermal Conductivity and Electrical Insulation Properties
Source: Polymers (Basel). 2024 Jul 27;16(15):2139. doi: 10.3390/polym16152139 (PMC11313851; doi:10.3390/polym16152139)
Supplement: Supplementary file 1 [file polymers-16-02139-s001.zip › polymers-3111768-supplementary.pdf]

Supporting Information

Constructing heterostructured MWCNT-BN hybrid fillers in hot-pressed electrospun TPU composites: Achieving superior thermal conductivity and electrical insulation properties

Yang Zhang[1], Shichang Wang[1], Hong Wu[*], Shaoyun Guo

State Key Laboratory of Polymer Materials Engineering, Sichuan Provincial Engineering Laboratory of Plastic/Rubber Complex Processing Technology, Polymer Research Institute of Sichuan University, Chengdu 610065, China

[*] Corresponding Author, E-mail: wh@scu.edu.cn (H. Wu).

[1] The authors have equal contribution to this work.

Result and discussion

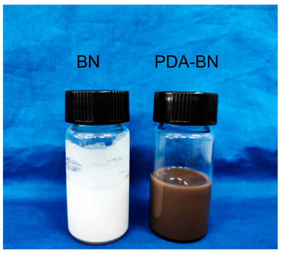

Figure S1. Optical images of BN and PDA-BN.

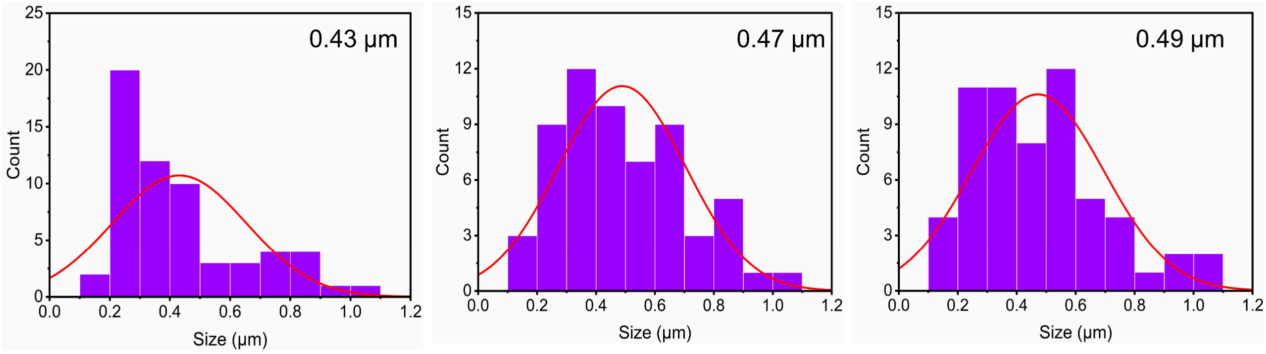

Figure S2. Lateral size statistics of BN, PDA-BN and MWCNT-BN.

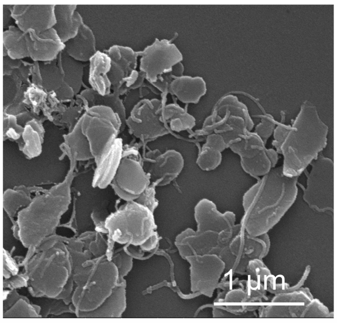

Figure S3. SEM image of MWCNT-BN.

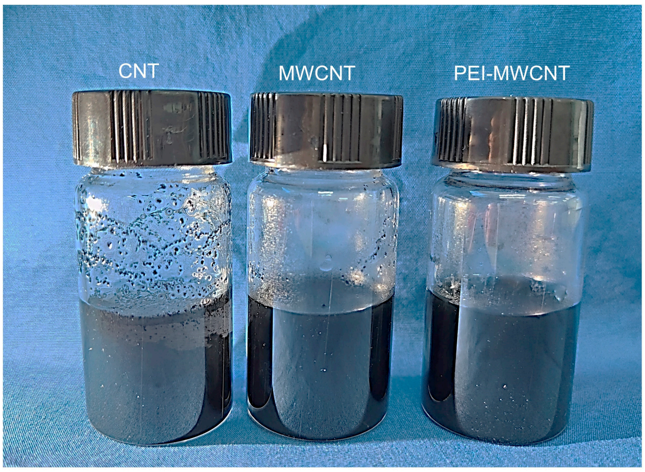

Figure S4. Optical images of CNT, MWCNT and PEI-MWCNT

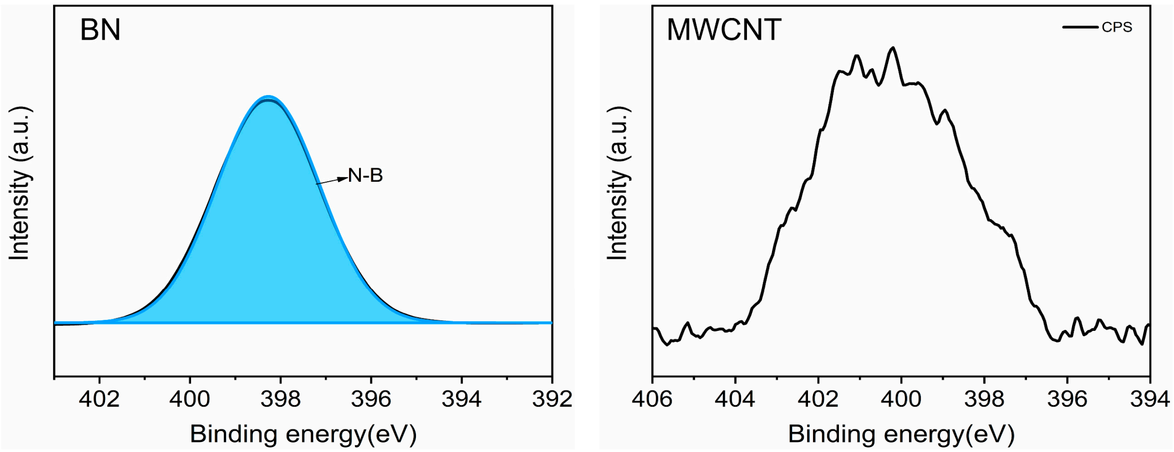

Figure S5. N1s XPS core level of BN and MWCNT.

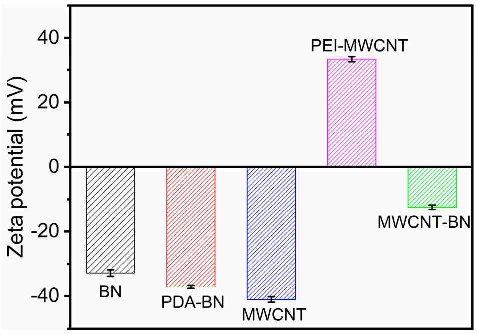

Figure S6. Zeta potential of BN, PDA-BN, MWCNT, PEI-MWCNT and MWCNT-BN.

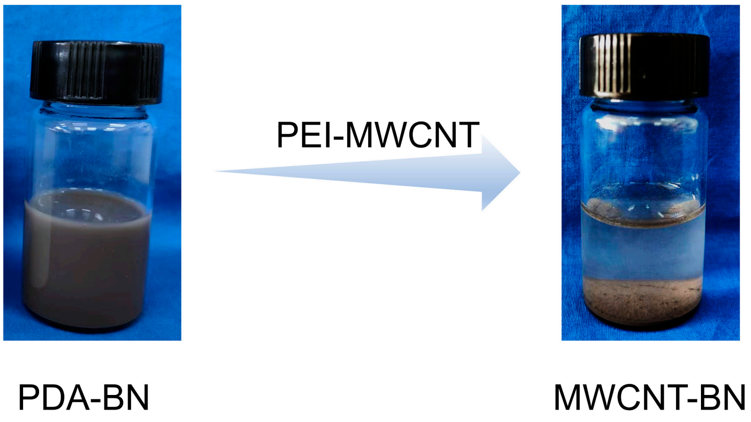

Figure S7. Optical images the fabrication process of MWCNT-BN.

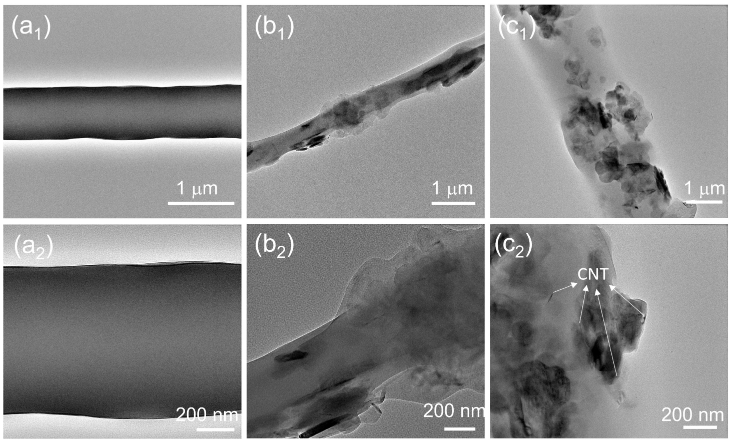

Figure S8. TEM images of (a) TPU, (b) TPU/40BN and (c) TPU/40MWCNT-BN electrospun fibers, respectively.

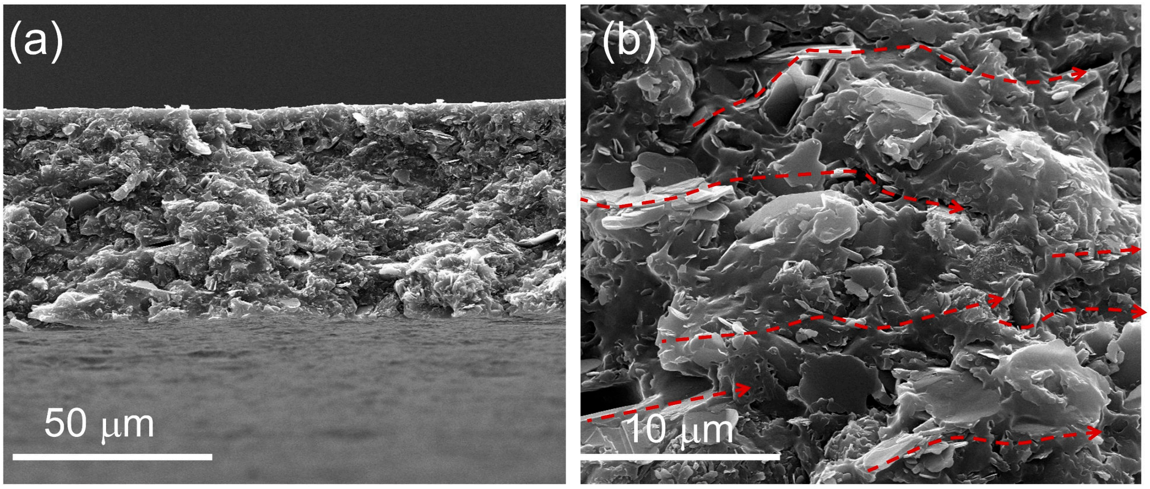

Figure S9. Cross-sectional morphologies of the TPU/40MWCNT-BN composite film.

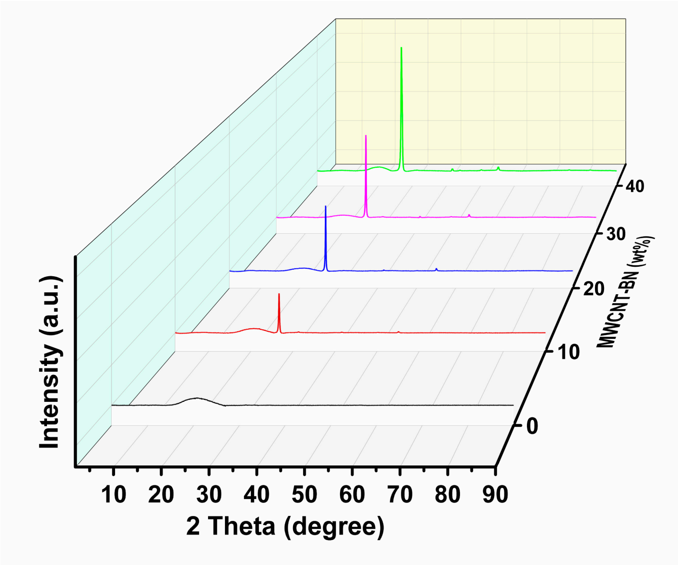

Figure S10. XRD patterns of TPU/MWCNT-BN composite film with different filler content.

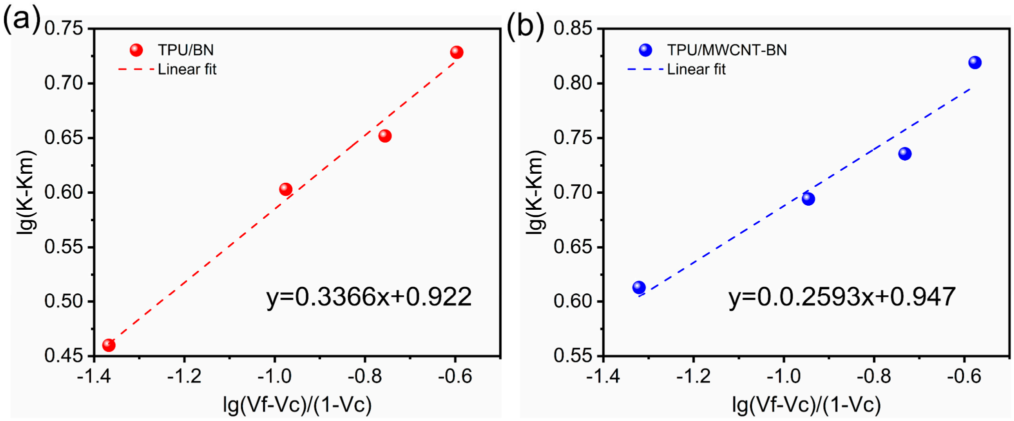

Figure S11. Foygel model fitting curves of composite fims: (a) TPU/BN; (b) TPU/MWCNT-BN.

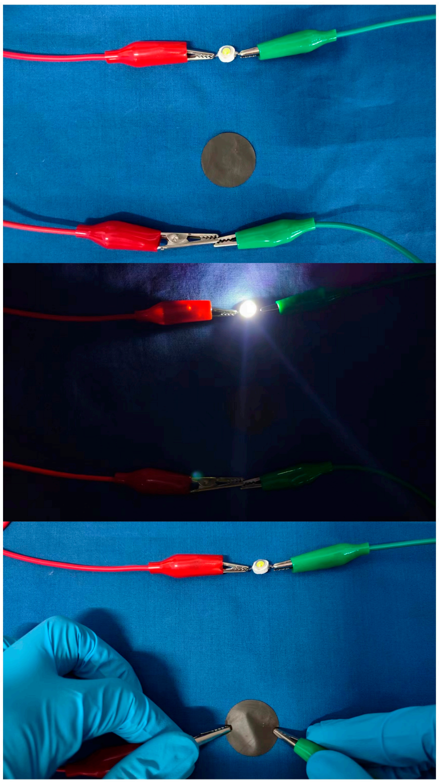

Figure S12. Insulation exhibition of TPU/40MWCNT-BN compsoite films.

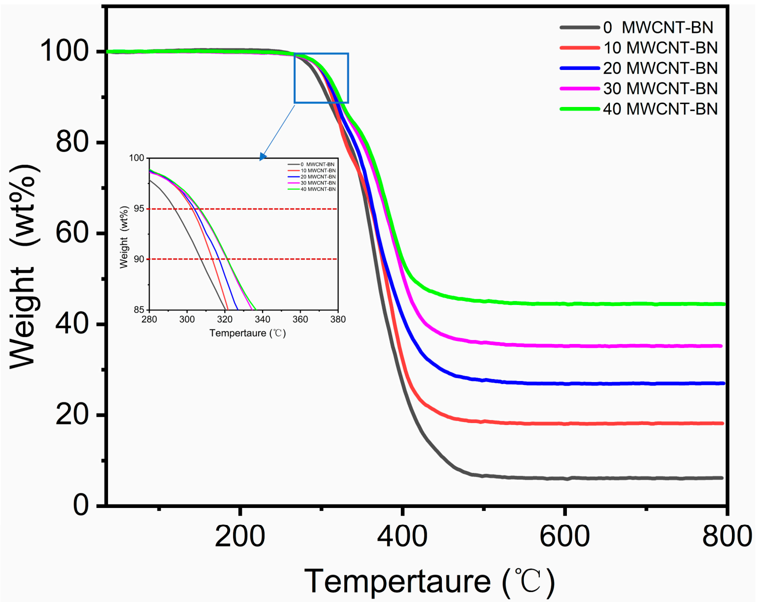

Figure S13. TGA curves of TPU/MWCNT-BN composite film with different filler content.

Table S1. Atomic weight ratio of BN, PDA-BN, MWCNT, PEI-MWCNT and MWCNT-BN

Elemental analysis（%）				
**Sample**	**C**	**N**	**O**	**B**
BN	18.58	38.27	5.3	37.84
PDA-BN	55.92	15.69	14.68	13.71
MWCNT	93.42	0.6	5.98	0
PEI-MWCNT	87.51	6.15	6.35	0
MWCNT-BN	43.35	24.38	10.70	21.56

Table S2. Comparison of in-plane TC of TPU/MWCNT-BN composite films and other thermally conductive composite reported previously.

Filler	Matrix	Loading	TC (W/mK)	Reference
BN	EP	50 wt%	1.957	1 (2022)
BN/MWCNT	PDMS	32 wt%	4.28	2 (2022)
BN	EP	21.8 wt%	6	3 (2022)
GF-BN	PI	30 wt%	2.532	4 (2022)
BNNS	EP	20 wt%	0.88	5 (2022)
BN/CNT	PC	32 wt%	2.05	6 (2022)
BN	PC	30 wt%	3.09	7 (2018)
BN/GO	PP	50 wt%	5.01	8 (2021)
BNNS	SR	30 wt%	5.47	9 (2015)
BNNS	EP	40 wt%	5.86	10 (2020)
BN	EP	40 wt%	6	11 (2014)
BN/CNT	PVDF	22.5 wt%	2.18	12 (2019)
BN	PI	30 wt%	2.81	13 (2019)
BNNS	PVDF	4 wt%	4.69	14 (2018)

Table S3. Agari model fitting parameters of composite films.

Samples	C_1	C_2
TPU/BN	4.836	0.248
TPU/MWCNT-BN	6.405	0.287

Table S4. Foygel model fitting parameters of composite films.

Samples	β	K_0	R(K/W)
TPU/BN	0.3366	8.352	1.17×10^6
TPU/MWCNT-BN	0.2592	8.857	7.43×10^5

The specific calculation process of interface thermal resistance:

The mass fraction of the filler is transformed into the volume fraction of the filler, which is as follows:

$$\varphi_f = \frac{V_{BN}+V_{MWCMT}}{V_{TPU}+V_{BN}+V_{MWCNT}} = \frac{\frac{\omega_{BN}}{\rho_{BN}}+\frac{\omega_{MWCMT}}{\rho_{MWCNT}}}{\frac{\omega_{TPU}}{\rho_{TPU}}+\frac{\omega_{BN}}{\rho_{BN}}+\frac{\omega_{MWCNT}}{\rho_{MWCNT}}} \quad (1)$$

For TPU/BN composite films:

$$\varphi_f = \frac{\frac{\omega_f}{\rho_{BN}}}{\frac{1-\omega_f}{\rho_{TPU}}+\frac{\omega_f}{\rho_{BN}}} \quad (2)$$

For TPU/MWCNT-BN composite films:

$$\varphi_f = \frac{\frac{\frac{15}{16}\omega_f}{\rho_{BN}}+\frac{\frac{1}{16}\omega_f}{\rho_{MWCNT}}}{\frac{1-\omega_f}{\rho_{TPU}}+\frac{\frac{15}{16}\omega_f}{\rho_{BN}}+\frac{\frac{1}{16}\omega_f}{\rho_{MWCNT}}} \quad (3)$$

Where φ_f and ω_f are the volume fraction and mass fraction of the filler, respectively. ω_{TPU}, ω_{BN} and ω_{MWCNT} are the mass fraction of TPU, BN and MWCNT, respectively. V_{TPU}, V_{BN}, and V_{MWCNT} are the volume fraction of TPU, BN and MWCNT respectively. The density of TPU (ρ_{TPU}), BN (ρ_{BN})and MWCNT (ρ_{MWCNT}) are 1.23g/cm^3, 2.21 g/cm^3 and 2.29 g/cm^3, respectively.

Foygel model is defined as follow formula:

$$K-K_m=K_0[(V_f-V_c)/(1-V_c)]^{t(a)} \quad (4)$$

where K is in-plane TC of the TPU composite films; K_m is the in-plane TC of pure

TPU film; K_0 represents the pre-exponential factor depending on the filler; t(a) suggests that the TC index that related to the width (L) and thickness (D) ratio (a =L/D) of BN or MWCNT-BN, and the thickness of BN and MWCNT-BN is about 10 nm observed by AFM; V_f is the volume fraction of the filler; V_c represents the critical volume fraction of the filler under the thermal seepage threshold, which can be calculated by the following formula:

$$V_c(a>>1)=\frac{0.60}{a} \qquad (5)$$

The values of K_0 and t(a) can be successfully obtained through fitting the in-plane TC of TPU composite films. Based on this, the value of the interfacial thermal resistance (R) of TPU composite films can be calculated from the following formula:

$$R=\frac{1}{K_0 L (V_c)^{t(a)}} \qquad (6)$$
